# Association Between Suicide Donors and Outcomes in Heart Transplantation: A Retrospective Cohort Study

**DOI:** 10.21470/1678-9741-2025-0312

**Published:** 2026-09-08

**Authors:** Abdullah Saad, Aisha Imran, Fatima Siddiqui, Laiba Abbas

**Affiliations:** 1 Sindh Medical College and Jinnah Postgraduate Medical Centre, Karachi, Pakistan; 2 Dow Medical College, Karachi, Sindh, Pakistan; 3 Karachi Medical and Dental College, Karachi, Sindh, Pakistan

Dear Editor,

We commend Perazzo et al. for their timely investigation on the use of suicide donors in heart transplantation, a topic that has received limited attention despite its ethical and clinical importance^[^1^]^. Their finding of higher 30-day mortality in hearts from suicide donors raises important questions, particularly regarding the potential mechanisms and confounding factors that may influence outcomes. However, we note several limitations that may temper the interpretation of these results.

First, the small subgroup size (n = 6 suicide donors) restricts statistical power and inflates the risk of spurious associations. Comparisons of rare events between such unequal groups (six *vs.* 91) may overestimate the risk, and the absence of confidence intervals limits the reader’s ability to judge precision. As Concato et al. emphasized, the strength of observational data lies not in statistical significance alone but in careful control for bias and variability^[^2^]^.

Second, no multivariate analysis was performed to adjust for potential confounders, such as ischemia time, cardiopulmonary bypass duration, or recipient illness severity. Given the established influence of these perioperative factors on graft outcomes, omission of adjusted analyses raises the possibility that the observed differences may be attributable to baseline imbalances rather than donor mechanism per se^[^3^]^.

The authors reported higher mortality rates for hearts from suicide donors without stratifying by mechanism of death, despite evidence that livers and lungs from suicide donors generally function normally compared to those from other causes of brain death. Hoti et al. demonstrated comparable outcomes in liver transplantation using grafts from hanging donors^[^4^]^, and Mohite et al. reported similar findings for lungs from hanging donors^[^5^]^. This suggests a logical gap: if these organs perform well despite suicide, it is possible that specific mechanisms (such as hanging-related hypoxia or overdose-related intoxication) or perioperative complications have a greater impact on cardiac outcomes than suicide itself. Attributing worse outcomes solely to suicide risks oversimplifies the relationship and may unnecessarily limit the donor pool if these factors are not considered.

Beyond these methodological aspects, we note that the study excluded eight patients due to incomplete data. While this is a reasonable approach, future analyses could benefit from sensitivity testing or multiple imputation methods to assess the impact of missing data and minimize the exclusion bias. Likewise, the single-center design, while allowing for consistent protocols, may limit the generalizability to other transplant programs with differing donor selection practices or perioperative strategies. Expansion to multicenter or registry-based cohorts would help determine whether the observed associations are reproducible across diverse populations.

The follow-up period is another important consideration. The current report is limited to 30-day outcomes, which are critical for early postoperative safety but do not capture long-term graft survival, rejection episodes or functional status. Future research should aim to include medium- and long-term endpoints, as these are central to patient and graft prognosis after heart transplantation.

Finally, while the discussion offers interesting hypotheses regarding psychological essentialism and immune dysregulation, these interpretations should be approached with caution. Without supporting biomarker data, they risk reinforcing the stigma toward suicide victims and their families. A more balanced discussion of the ethical and practical considerations of using suicide donor organs would have been welcome, particularly given the existing literature cautioning against perpetuating myths that may inadvertently reduce organ utilization^[^6^]^.

In conclusion, Perazzo et al. initiated an important discussion on the use of suicide donors for heart transplantation. Their findings are thought-provoking but should be interpreted with caution, given the small sample size, limited follow-up, and absence of multivariate adjustment. Future studies should ideally be multicenter, include longer-term outcomes, and stratify donors by the mechanism of suicide and toxicology findings. Incorporating biomarker data could also help clarify whether biological factors contribute to the observed differences. Equally important is framing this debate with ethical sensitivity to avoid reinforcing the stigma while ensuring that potentially viable donor hearts are not overlooked. By addressing these aspects, future research can strengthen the evidence base and guide fair and scientifically grounded donor selection policies.
